# A multiparametric [^18^F]FDG PET/MRI diagnostic model including imaging biomarkers of the tumor and contralateral healthy breast tissue aids breast cancer diagnosis

**DOI:** 10.1007/s00259-019-04331-6

**Published:** 2019-06-13

**Authors:** Doris Leithner, Joao V. Horvat, Blanca Bernard-Davila, Thomas H. Helbich, R. Elena Ochoa-Albiztegui, Danny F. Martinez, Michelle Zhang, Sunitha B. Thakur, Georg J. Wengert, Anton Staudenherz, Maxine S. Jochelson, Elizabeth A. Morris, Pascal A. T. Baltzer, Paola Clauser, Panagiotis Kapetas, Katja Pinker

**Affiliations:** 1 Department of Radiology, Breast Imaging Service, Memorial Sloan Kettering Cancer Center, 300 E 66th St, 7th Floor, New York, NY 10065 USA; 2 Department of Diagnostic and Interventional Radiology, University Hospital Frankfurt, Frankfurt, Germany; 3 Department of Epidemiology and Biostatistics, Memorial Sloan Kettering Cancer Center, New York, NY USA; 4 Department of Biomedical Imaging and Image-guided Therapy, Division of Molecular and Gender Imaging, Medical University Vienna, Vienna, Austria; 5 Department of Biomedical Imaging and Image-guided Therapy, Division of Nuclear Medicine, Medical University of Vienna, Vienna, Austria

**Keywords:** Breast cancer, Positron emission tomography, Fluorodeoxyglucose, Magnetic resonance imaging, Imaging biomarker

## Abstract

**Purpose:**

To develop a multiparametric [^18^F]FDG positron emission tomography/magnetic resonance imaging (PET/MRI) model for breast cancer diagnosis incorporating imaging biomarkers of breast tumors and contralateral healthy breast tissue.

**Methods:**

In this prospective study and retrospective data analysis, 141 patients (mean 57 years) with an imaging abnormality detected on mammography and/or ultrasound (BI-RADS 4/5) underwent combined multiparametric [^18^F]FDG PET/MRI with PET/computed tomography and multiparametric MRI of the breast at 3 T. Images were evaluated and the following were recorded: for the tumor, BI-RADS descriptors on dynamic contrast-enhanced (DCE)-MRI, mean apparent diffusion co-efficient (ADCmean) on diffusion-weighted imaging (DWI), and maximum standard uptake value (SUVmax) on [^18^F]FDG-PET; and for the contralateral healthy breast, background parenchymal enhancement (BPE) and amount of fibroglandular tissue (FGT) on DCE-MRI, ADCmean on DWI, and SUVmax. Histopathology served as standard of reference. Uni-, bi-, and multivariate logistic regression analyses were performed to assess the relationships between malignancy and imaging features. Predictive discrimination of benign and malignant breast lesions was examined using area under the receiver operating characteristic curve (AUC).

**Results:**

There were 100 malignant and 41 benign lesions (size: median 1.9, range 0.5–10 cm). The multivariate regression model incorporating significant univariate predictors identified tumor enhancement kinetics (*P* = 0.0003), tumor ADCmean (*P* < 0.001), and BPE of the contralateral healthy breast (*P* = 0.0019) as independent predictors for breast cancer diagnosis. Other biomarkers did not reach significance. Combination of the three significant biomarkers achieved an AUC value of 0.98 for breast cancer diagnosis.

**Conclusion:**

A multiparametric [^18^F]FDG PET/MRI diagnostic model incorporating both qualitative and quantitative parameters of the tumor and the healthy contralateral tissue aids breast cancer diagnosis.

## Introduction

In recent years, positron emission tomography (PET) and multiparametric magnetic resonance imaging (mpMRI) of the breast have been established as promising tools [1–3] that provide morphologic and functional data and are of complementary value for the differentiation of benign and malignant breast tumors [4]. MpMRI consisting of dynamic contrast-enhanced MRI (DCE-MRI) [5, 6] and diffusion-weighted imaging (DWI) [7, 8] provides information on tumor neoangiogenesis, microenvironment and microstructural changes. PET using the radiotracer ^18^-fluorodeoxyglucose ([^18^F]FDG) provides data on lesion glucose metabolism and is of complimentary value to MRI for breast cancer diagnosis [2, 9–13]. To combine the strengths of these imaging techniques, hybrid imaging systems have been developed and introduced into the clinical routine. With PET/MRI systems, morphologic and different functional information can be merged to non-invasively characterize breast tumors as benign or malignant.

The predictive and prognostic value of imaging features derived from healthy breast tissue such as background parenchymal enhancement (BPE) in DCE-MRI have been recognized only recently. BPE is the physiological contrast enhancement of fibroglandular tissue (FGT). Previous studies have found correlations between higher levels of BPE and FGT and increased cancer risk in a high risk population [14, 15], while associations between apparent diffusion coefficient (ADC) values of normal breast tissue and cancer outcomes have not been explored. Similar to MRI, healthy breast parenchyma shows varying degrees of tracer uptake in [^18^F]FDG PET, reflecting its physiologic activity, referred to as breast parenchymal uptake (BPU) [16, 17] and is directly correlated with BPE and FGT. It has therefore been suggested that BPU could potentially serve as another breast cancer imaging biomarker [16–18].

With the clinical implementation of PET/MRI scanners and given the improvements in diagnosis and prognosis that can be achieved with mpMRI, it is of interest to explore the potential of combined [^18^F]FDG PET/MRI to improve diagnostic accuracy and obviate unnecessary breast biopsies of benign breast tumors detected with MRI. To date it is unclear which quantitative and qualitative imaging features from both tumor and healthy tissue contribute to an accurate diagnosis. To close this gap in knowledge we aimed to develop a multiparametric [^18^]FDG PET/MRI model for breast cancer diagnosis incorporating imaging biomarkers of breast tumors and contralateral healthy breast tissue.

## Material and methods

The local institutional review board (Ethikkommission Medizinische Universitaet Wien/Ethics Committee Medical University of Vienna) approved this prospective single-institution study (EK 510/2009) and retrospective data analysis. The research was performed in accordance with relevant guidelines/regulations and informed consent was obtained from all patients prior to [^18^F]FDG PET/MRI.

### Patient population

Patients were examined with [^18^F]FDG PET/computed tomography (CT) and mpMRI in a prospective trial. For the current study that focused on model development, the prospectively populated research database was searched for patients who underwent multiparametric [^18^F]FDG PET/MRI with PET/CT and mpMRI of the breast at 3 T between December 2009 and September 2014 and who met the following inclusion criteria: 18 years or older; not pregnant; not breastfeeding; no previous breast cancer treatment; imaging abnormality on mammography or breast ultrasound (BI-RADS 4, suspicious abnormality; or 5, highly suggestive for malignancy); and no contraindications for MRI or MRI contrast agents. Exclusion criteria were high-risk patients (confirmed BRCA 1/2 mutation, lifetime risk >20%, incomplete examinations, severe motion or susceptibility artifacts, no histopathologic confirmation by image-guided or surgical biopsy after MRI and [^18^F]FDG PET/CT, and presence of another suspicious lesion in the contralateral breast. Thus, 141 patients with a unilateral breast lesion in mammography, ultrasound, MRI, and [^18^F]FDG PET/MRI (BI-RADS 1) were included for analysis. Electronic medical records were reviewed to record patient age and histopathology results, including tumor grade, subtype, and receptor status for malignant lesions. A number of patients included in this study have been analyzed and reported before in different contexts [4, 11, 16].

### Imaging

All patients underwent combined multiparametric [^18^F]FDG PET/MRI with PET/CT using the tracer [^18^F]FDG and multiparametric MRI of the breast at 3 T. Examinations were scheduled no longer than 6 days apart (mean, 1.15; range, 0–6; same day, *n* = 70; 1 day, *n* = 31; 2 days, *n* = 12; 3 days, *n* = 11; 4 days, *n* = 11; 5 days, *n* = 5; 6 days, *n* = 1).

#### [18F]FDG pet/CT

A combined PET/CT in-line system (Biograph 64 TruePoint PET/CT system, Siemens, Erlangen, Germany) was used to acquire PET images. Patients fasted 6 h before they were injected with approximately 300 MBq [^18^F]FDG, depending on the patient’s weight, with an uptake time of 45 min. Blood glucose levels were below 150 mg/dl (8.3 mmol/l) at the time of tracer application. A prone PET dataset of the breasts was acquired and an unenhanced low-dose CT was used for attenuation correction. PET images were reconstructed using the TrueX algorithm (Siemens, Erlangen, Germany), which includes commonly used correction factors, as well as a specific point-spread function correction. Four iterations per 21 subsets were used, with a matrix size of 168 × 168, trans-axial field of view of 605 mm (pixel size, 3.6 mm), and section thickness of 5 mm.

#### Multiparametric MRI

MRI was performed in the prone position using a 3 T MRI scanner (Tim Trio, Siemens, Erlangen, Germany) and a four-channel breast coil (InVivo, Orlando, FL, USA). In premenopausal patients, MRI was performed between the 7th and 14th day of the menstrual cycle to minimize hormonal influence on BPE [19]. All patients underwent a state-of-the-art MRI protocol as follows.

T2-weighted turbo spin echo (TSE) imaging with fat suppression: repetition time/echo time (TR/TE) 4800/9 ms; field of view (FOV) 340 mm; 48 slices at 3 mm; flip angle 128°; matrix 384 × 512; time of acquisition (TA) 2 min, 16 s.

Axial three-acquisition trace diffusion-weighted, double-refocused single-shot echo-planar imaging (EPI) with inversion recovery fat suppression: TR/TE/time of inversion (TI) 8000/59/210 ms; FOV 360 × 202 mm; 24 slices at 5 mm; intersection gap (%) 10; matrix 172 × 96 [50% oversampling]; b-values 50, 850 s/mm; TA 2 min, 56 s [20].

DCE-MRI using a split-dynamics protocol until 12/2011 [21] consisting of five alternating sections of high-spatial and high-temporal resolution T1-weighted sequences: (1) high spatial resolution unenhanced coronal T1-weighted turbo fast-low-angle-shot-(FLASH)-3D sequences without preparation pulse, with selective water-excitation (TR/TE 877/3.82 ms; FOV 320 mm; 96 slices; 1 mm isotropic; matrix 320 × 134; one average; bandwidth 200 Hz/pixel; TA 2 min); (2) high temporal resolution coronal T1-weighted Volume Interpolated Breath-hold Examination (VIBE) (TR/TE 3.61/1.4 ms; FOV 320 mm; 72 slices; 1.7 mm isotropic; matrix 192 × 192; one average; bandwidth 400 Hz/pixel; 13.2 s per volume; (3) repeated high spatial resolution unenhanced coronal T1-weighted 3D-FLASH at peak contrast enhancement; (4) repeated high temporal resolution VIBE (25 measurements), repeated 3D-FLASH, TA 5 min, 35 s); and (5) high spatial resolution T1-weighted images 3D-FLASH for delayed phase. Total TA was 9 min 20 s.

DCE-MRI protocol from 01/2012 onwards: transversal T1-weighted time-resolved angiography with stochastic trajectories (TWIST); water excitation fat-saturation; TR/TE 6.23/2.95 ms; flip angle 15°, FOV 196 × 330 mm^2^; 144 slices; spatial resolution 0.9 × 0.9 × 1 mm; temporal interpolation factor 2; temporal resolution 14 s; matrix 384 × 384; one average; center k-space region, resampling rate of 23%; reacquisition density of peripheral k-space 20%; TA 6 min, 49 s.

DCE-MRI image series were acquired before and after the injection of a standard dose (0.1 mmol/kg body-weight) of Gadotaremeglumine (Gd-DOTA; Dotarem®, Guerbet, France) as a bolus using a power injector (Spectris Solaris EP®, Medrad, Pittsburgh, PA, USA).

MpMRI and PET data were fused semi-automatically using the “landmark matching” tool of the TrueD fusion workstation (Siemens, Erlangen, Germany) to generate combined [^18^F]FDG PET/MRI data.

### Data analysis

#### [18F]FDG pet

All measurements were performed by a radiologist (D.L., 3 years of experience) trained in hybrid imaging under the supervision of a nuclear medicine physician. A three-dimensional volume of interest (VOI) was placed around the primary breast tumor to record lesion maximum standardized uptake value (SUVmax) using the TrueD fusion workstation (Siemens, Erlangen, Germany). The VOI was established using the Region Grow function with a fixed threshold as determined by the reader to represent PET metabolic tumor volume but excluding physiologic [^18^F]FDG uptake in surrounding tissues. For quantification of BPU of the normal contralateral breast, a VOI was placed around the glandular tissue. Adequate distance to the nipple and areola was kept. All VOI measurements were repeated three times and averaged to ensure data consistency.

#### DCE-MRI

DCE-MRI data was evaluated by two radiologists in consensus (K.P., 12 years of experience, and D.L., 3 years of experience). Both readers were aware that the patient had a lesion but were not provided with previous imaging or histopathology results. All images were assessed using the revised ACR MRI BI-RADS lexicon [22]. Lesions were classified as mass or non-mass-enhancement (NME), and described accordingly using BI-RADS descriptors. For the evaluation of enhancement kinetics, automated semi-quantitative curve-type analysis was performed using dedicated software (DCE tool plugin v2.2 for OSIRIX software) [23]. The largest diameters measured on DCE-MRI were recorded. For the contralateral healthy breast, BPE and FGT were assessed qualitatively, as recommended by the revised ACR MRI BI-RADS lexicon [22]. BPE was evaluated using DCE-MR images 90 s after contrast agent administration and classified as minimal, mild, moderate, or marked. FGT was evaluated using unenhanced T1-weighted fat-suppressed images and graded as ACR (a) for almost entirely fatty breasts, (b) for scattered fibroglandular tissue, (c) for heterogeneous fibroglandular tissue, and (d) for extreme amount of fibroglandular tissue. Both BPE and FGT were assessed in the healthy contralateral breast only.

#### DWI

DCE-MR images were used to identify the lesion on DWI with ADC mapping. The slice with the greatest diameter of the tumor was selected, and an ROI (minimum 10 mm^2^) was drawn on the part of the tumor with the lowest ADC by two radiologists in consensus (K.P., and D.L.). ADCmean was recorded. For quantification of ADCmean of the healthy contralateral breast, a 10 mm^2^ standardized 2D ROI was manually drawn in normal appearing fibroglandular tissue. For each lesion and healthy breast, ROI measurements were repeated three times and results were averaged.

All tumor characteristics on [^18^F]FDG PET, DCE-MRI and DWI are displayed in Table 1, while parameters derived from healthy breast tissue can be found in Table 2.

**Table 1:** Lesion imaging characteristics assessed on [^18^F]FDG PET/MRI

Imaging modality	BI-RADS descriptors/imaging characteristics		Categories/explanation
DCE-MRI	Mass	Shape	Round, oval, irregular
		Margin	Circumscribed, irregular, spiculated
		Internal enhancement characteristics	Homogeneous, heterogenous, rim enhancement, dark internal septations
	NME	Distribution	Focal, linear, segmental, regional, multiple regions, diffuse
		Internal enhancement pattern	Homogeneous, heterogeneous, clumped, clustered ring
		Symmetry	Symmetric, asymmetric
	Kinetic curve	Initial phase	Slow, medium, fast
		Delayed phase	Persistent, plateau, washout
DWI	ADCmean		Mean ADC value with ROI drawn over the part of the lesion with the visually lowest ADC
[^18^F]FDG PET	SUVmax		SUVmax value with ROI drawn over the whole tumor

*NME* non mass enhancement, *ADC* apparent diffusion coefficient, *SUVmax* maximum standardized uptake value

**Table 2:** Imaging characteristics of the healthy contralateral breast assessed on [^18^F]FDG PET/MRI

Imaging modality	Imaging characteristics	Categories/explanation
DCE-MRI	BPE	Minimal, mild, moderate, marked
	FGT	Almost entirely fatty, scattered fibroglandular tissue, heterogeneous fibroglandular tissue, extreme amount of fibroglandular tissue
DWI	ADCmean	Mean ADC value of healthy breast tissue with ROI drawn over the whole parenchyma
[^18^F]FDG PET	BPU	Tracer uptake of normal healthy breast parenchyma quantified by SUVmax

*BPE* background parenchymal enhancement, *FGT* amount of fibroglandular tissue, *ADC* apparent diffusion coefficient, *BPU* breast parenchymal uptake

### Histopathology

Histopathology served as standard of reference. Histopathological diagnosis was established using tissue derived from image-guided needle biopsy or surgical specimens. In case of a high-risk lesion with uncertain potential for malignancy, the final diagnosis was established with surgery.

In 33 patients with benign lesions, final histology was established via core-needle biopsy, while three patients opted for elective surgery. Five high-risk lesions on breast biopsy remained benign on surgery. In 48 patients with malignant lesions, final histology was established from surgical specimen, while in 52 patients that received neoadjuvant chemotherapy, histology was obtained from core-needle biopsy specimen.

### Statistical analysis

Statistical tests were performed using SAS, v9.4 (SAS Institute, Cary, NC, United States). All calculations were performed on a per-lesion basis. The main outcome was presence of malignancy. Using histopathology as the standard of reference, disease status was dichotomized as positive or negative for malignancy. Dichotomized variables further included mass shape (round/oval/no enhancement vs. irregular), mass margins (circumscribed vs. irregular/spiculated), mass internal enhancement characteristics (homogeneous/dark internal septation vs. heterogeneous/rim enhancement), NME distribution (region/segmental vs. multiple regions/diffuse), and NME pattern of enhancement (homogeneous vs. heterogeneous/clumped). Enhancement kinetics of the tumor (persistent, plateau, wash-out) were examined as a three-level categorical variable, while BPE (minimal, mild, moderate, marked) and FGT (ACR a–d) were assessed as four-level categorical variables. Patient age, lesion size, tumor and parenchyma ADCmean, and FDG SUVmax were kept in continuous form.

Univariate analysis, bivariate analysis and multivariate logistic regression were applied to assess the relationships between malignancy and imaging features. We analyzed the frequency distribution of demographic, clinical and imaging characteristics using the chi-square test or Fisher’s exact test for categorical variables and quantile-quantile plots (Q–Q plots), non-parametric Wilcoxon test and T-test for continuous variables, where appropriate.

Variables with an association in bivariate analysis were examined. We assessed the association between histology and the following imaging features: BPE of the healthy breast, internal enhancement characteristics, mass margin and shape, enhancement kinetics, tumor ADCmean, tumor SUVmax, and tumor size. A priori confounder, age, was also examined. Confounders were included in the final model if they altered beta estimates by more than 10%. Receiver operating characteristic (ROC) analysis was performed to examine predictive discrimination.

A stratified analysis was conducted to test confounding by menopause.

*P* values less than 0.05 were considered statistically significant.

## Results

There were 100 malignant and 41 benign lesions in 141 patients (140 female; mean age, 57 ± 14.3 years, range 18–86 years). Among these lesions, there were 121 masses (93 malignant, 28 benign) and 20 NME lesions (13 malignant, 7 benign) as defined according to the MRI BI-RADS lexicon. The average size of malignancies (27.3 mm) was larger than the size of benign lesions (22.6 mm) (*P* = 0.0227).

On DCE-MRI, morphological and kinetic features predictive of breast cancer presenting as masses were irregular/spiculated margin, irregular shape, heterogeneous internal enhancement and plateau/wash-out curves (*P* < 0.0001). Likewise, frequent features for benign mass lesions were round/oval shape, circumscribed margins, homogeneous/dark septations internal enhancement characteristics and persistent enhancement curves (P < 0.0001). Patients with a malignancy showed decreased BPE of the contralateral healthy breast (*P* = 0.001), while a borderline significant difference was observed for FGT (*P* = 0.0564).

On DWI, malignancies showed significant lower average ADCmean (0.96 × 10^−3^ mm^2^/s) compared with benign lesions (1.52 × 10^−3^ mm^2^/s) (*P* < 0.0001).

On [^18^F]FDG PET, breast cancers showed a higher FDG avidity (mean SUVmax 5.2) than benign tumors (mean SUVmax 2.6) (P < 0.0001).

Stratified analysis showed that menopause is not a confounder to our results.

Results of univariate analysis are summarized in Table 3.

**Table 3:** Results of univariate analysis: qualitative and quantitative variables for predicting malignancy

Total	Malignant (*n* = 100)	Benign (*n* = 41)	*P* value
Patient age			0.0007
Mean (SD) Median (min-max)	59.85 (12.01) 61.33 (36.88–86.11)	49.85 (17.00) 50.29 (0.0–86.11)	
Tumor size			0.0227
Mean (SD) Median (min-max)	27.26 (20.56) 19.5 (6.0–100.0)	22.56 (19.178) 13.0 (5–80)	
Mass margin			<.0001
Circumscribed Irregular/spiculated	3 (2.5) 90 (75.0)	23 (19.2) 3 (2.5)	
Mass shape			<.0001
Round/oval/no enhancement Irregular	15 (12.4) 78 (64.5)	23 (19.0) 5 (4.1)	
Mass internal enhancement characteristics			<.0001
Homogeneous/dark Heterogeneous/rim	15 (12.5) 78 (65.0)	21 (17.5) 6 (5.0)	
NME distribution			1
Regional/segmental Multiple/diffuse	1 (5.0) 6 (30.0)	3 (15.0) 10 (50.0)	
NME enhancement pattern			0.2487
Homogeneous Heterogeneous/clumped	0 (0) 7 (65.0)	4 (20.0) 9 (45.0)	
Enhancement kinetics			<.0001
Persistent Plateau Wash-out	4 (2.9) 44 (31.9) 52 (72.5)	21 (15.2) 14 (10.1) 3 (2.2)	
BPE healthy breast			0.001
Minimal Mild Moderate Marked	53 (37.6) 36 (25.5) 9 (6.4) 2 (1.4)	8 (5.7) 20 (14.2) 10 (7.1) 3 (2.1)	
FGT healthy breast			0.0564
ACR A ACR B ACR C ACR D	30 (21.4) 43 (30.7) 19 (13.6) 30 (5.7)	5 (3.6) 17 (12.1) 10 (7.1) 8 (3.6)	
ADCmean tumor			
Mean (SD) Median (min-max)	1515.17 (325.05) 1475.0 (883–2520)	961.57 (228.61) 945.5 (521–1616)	<.0001
ADCmean healthy breast			0.1779
Mean (SD) Median (min-max)	1698.5 (265.09) 1684.0 (1121–2398)	1770.37 (265.29) 1806 (1329–2369)	
SUVmax tumor			<.0001
Mean (SD) Median (min-max)	5.19 (4.41) 3.97 (1.1–25.62)	2.56 (1.45) 2.27 (0.4–6.9)	
BPU healthy breast			0.0816
Mean (SD) Median (min-max)	1.7597 (0.59) 1.59 (0.88–4.55)	1.9483 (0.646) 1.92 (0.88–3.96)	

*BPE* background parenchymal enhancement, *FGT* amount of fibroglandular tissue, *ADC* apparent diffusion coefficient, *SUVmax* maximum standardized uptake value, *BPU* breast parenchymal uptake

### Multiparametric PET-MRI model

The previously published ADCmean cut-off of ≤1.25 × 10^−3^ mm^2^/s was used to dichotomize ADC values as positive or negative to differentiate benign from malignant tumors [30]. Multivariate logistic regression analysis determined that tumor ADCmean in DWI, tumor enhancement kinetics, and BPE of the contralateral healthy breast in DCE-MRI were significantly associated with breast cancer diagnosis.

**Fig. 1:**
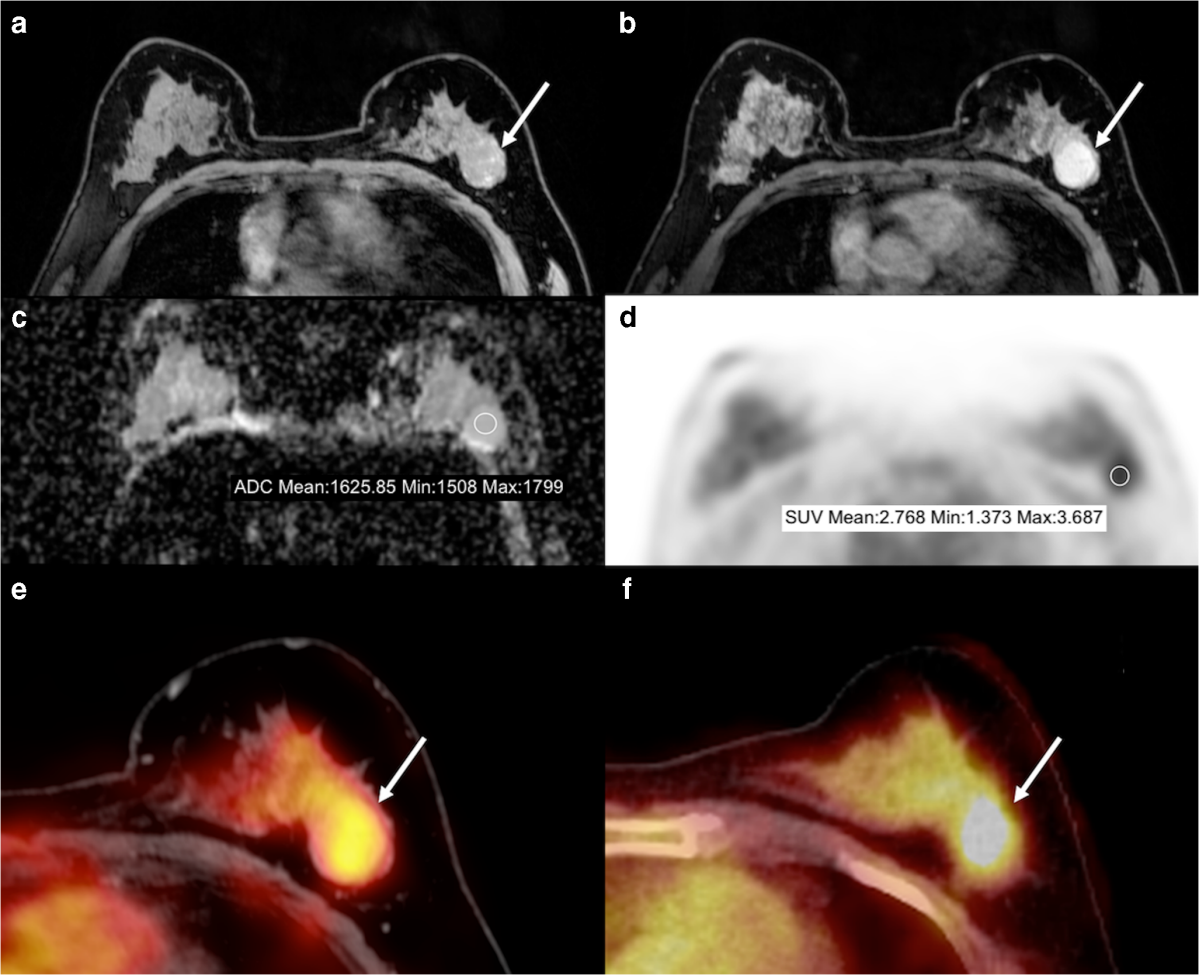
A 55-year-old postmenopausal woman with a fibroadenoma in the left breast presenting as a round, circumscribed mass with homogeneous enhancement on DCE-MRI. The lesion had no hindered diffusivity on DWI with an ADCmean of 1.625 10^−3^ mm^2^/s and was mildly [^18^F]FDG avid on PET with an SUVmax of 3.7. Unenhanced T1-weighted imaging shows heterogeneous fibroglandular tissue (ACR C) of the healthy breast, with moderate BPE on DCE-MRI. ADCmean of the healthy contralateral breast parenchyma is 1.642 10^−3^ mm^2^/s. On [^18^F]FDG PET, BPU of normal breast parenchyma is relatively high with an SUVmax of 3. **A** unenhanced fat-saturated T1-weighted MRI; **B** DCE-MRI; **C** DWI; **D** [^18^F]FDG PET; **E** fused [^18^F]FDG PET/MRI; **F** [^18^F]FDG PET/CT

**Fig. 2:**
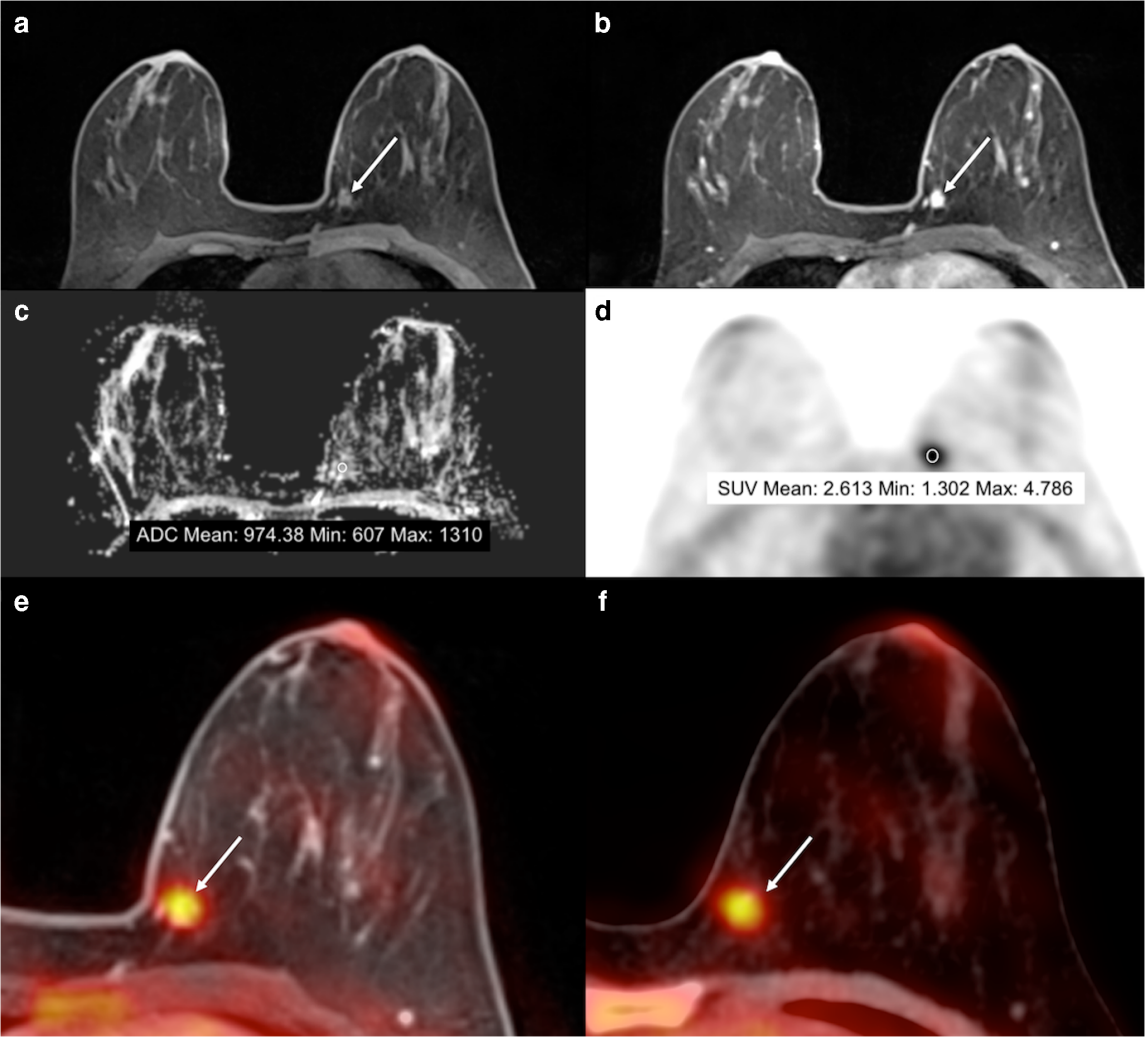
A 45-year-old premenopausal woman with an invasive ductal carcinoma in the left breast presenting as a mass lesion with irregular shape and margin and homogeneous contrast enhancement on DCE-MRI. The lesion displays hindered diffusivity on DWI with an ADCmean of 0.974 10^−3^ mm^2^/s and is highly [^18^F]FDG avid on PET with an SUVmax of 4.8. Unenhanced fat-saturated T1-weighted MR imaging shows a scattered amount of fibroglandular tissue (ACR B) in the healthy breast, while BPE on DCE-MRI is minimal. On DWI with ADC mapping, the ADC value of normal breast parenchyma is 1.693 10^−3^ mm^2^/s. On [^18^F]FDG-PET, BPU of healthy breast parenchyma is relatively low (SUVmax 1.9). **A** unenhanced fat-saturated T1-weighted MRI; **B** DCE-MRI; **C** DWI; **D** [^18^F]FDG PET; **E** fused [^18^F]FDG PET/MRI; **F** [^18^F]FDG PET/CT

**Fig. 3:**
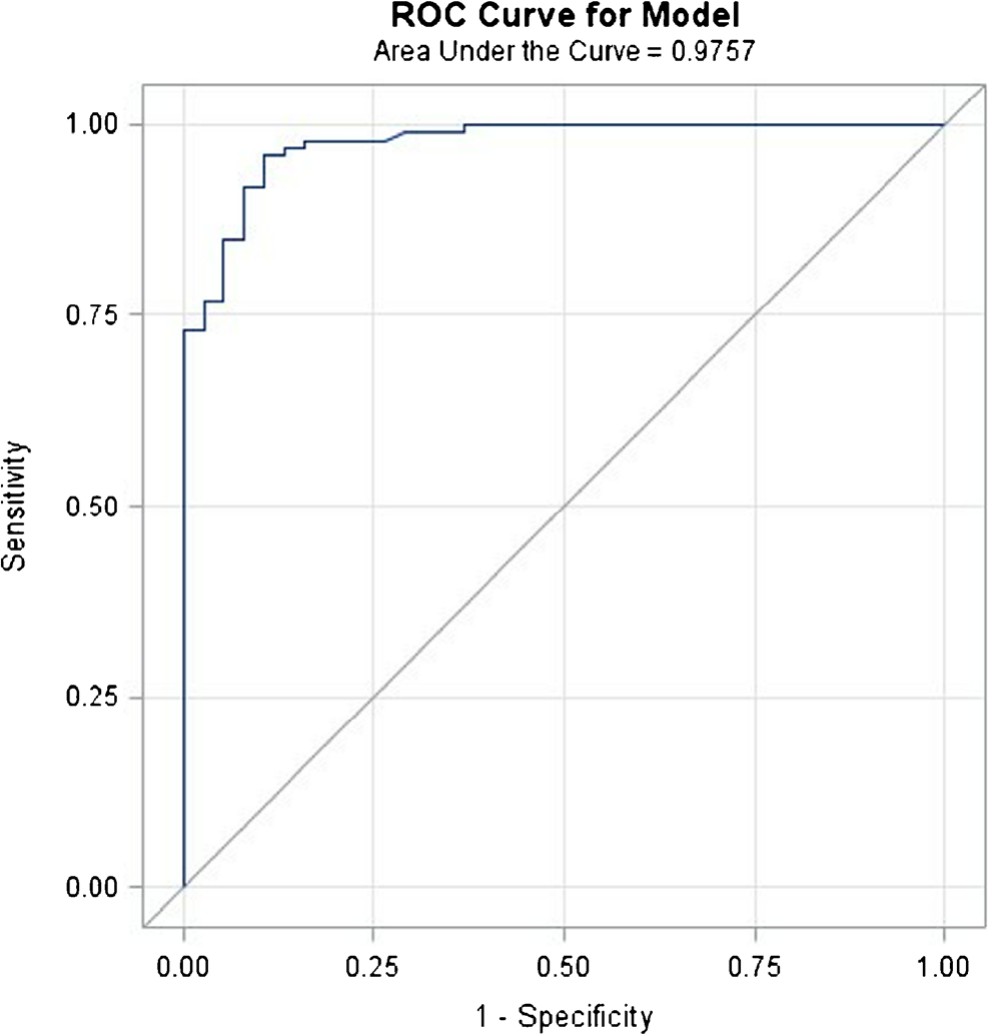
ROC curve for the developed multiparametric PET/MRI model for breast cancer diagnosis including tumor enhancement kinetics, tumor ADCmean and BPE of the contralateral healthy breast

Following these findings, our multiparametric [^18^F]FDG PET/MRI model incorporated these three significant univariate predictors. Based on this combination, the model discriminated between benign (Fig. 1) and malignant tumors (Fig. 2) with an area under the curve (AUC) value of 0.98 (Fig. 3).

The odds ratio (OR) of a lesion with ADCmean ≤1.25 × 10^−3^ mm^2^/s being a breast cancer is 1 (OR: 0.993, 95% confidence interval (CI): 0.99–0.996, *P* < 0.001). Lesions with a plateau or washout type kinetic curve had 18.7 greater odds of being malignant (OR: 18.7, 95%CI: 3.86–90.69) compared with those with persistent type enhancement (*P* = 0.0003). Lesions with decreased BPE of the contralateral healthy breast had 0.1 greater odds of being malignant (OR: 0.14, 95%CI: 0.04–0.49, *P* = 0.0019).

## Discussion

With the increasing use of PET/MRI worldwide, it is of interest to clarify whether qualitative and quantitative MRI and PET imaging biomarkers can contribute to accurate breast cancer diagnosis. We analyzed the contribution of tumor [^18^F]FDG SUVmax, BPU of healthy breast tissue, tumor DCE-MRI BI-RADS descriptors, BPE, and FGT of the contralateral healthy breast and ADCmean of the tumor and healthy breast tissue. The results of this study show that a multiparametric [^18^F]FDG PET/MRI diagnostic model incorporating both qualitative and quantitative parameters of the tumor and the healthy contralateral tissue aids breast cancer diagnosis. In particular, tumor enhancement kinetics, tumor ADCmean, and BPE of the contralateral healthy breast were most strongly associated with breast cancer. While our findings indicate that imaging biomarkers of healthy breast parenchyma can contribute to breast cancer diagnosis, [^18^F]FDG PET parameters did not add to accuracy.

To date, in breast imaging, regression models based on MRI have already been shown to clarify which parameters are necessary for breast cancer detection and staging. Knuttel et al. developed a predictive model incorporating DCE-MRI parameters including FGT for prediction of the ductal carcinoma in situ (DCIS) component associated with the primary invasive breast cancer [24]. DWI with ADC mapping was also demonstrated to be a useful addition to prediction models presented by Rahbar et al. for the differentiation between high grade and non-high grade DCIS [25], and by Cheeney et al. for the prediction of upgrade to malignancy at surgical excision [26]. However, to date, neither a multiparametric model approach has been applied to PET/MRI of the breast nor have imaging features of the tumor as well as the healthy contralateral breast tissue been incorporated for cancer diagnosis. In this respect this study is particularly relevant.

Prior studies showed a positive correlation between BPE, FGT with MRI and BPU with [^18^F]FDG PET and therefore suggested that BPU could potentially aid as another easily quantifiable imaging biomarker for breast cancer [16, 17]. In our study, both features derived from [^18^F]FDG PET, SUVmax of the tumor and BPU SUVmax of the contralateral healthy breast, did not significantly contribute to breast cancer diagnosis. At least with respect to tumor SUVmax, this is not entirely surprising as in prior studies the additional information provided by [^18^F]FDG PET was not as great as originally anticipated. The radiotracer [^18^F]FDG has a good sensitivity but limited specificity and there is an overlap with several types of benign breast diseases. The use of other PET tracers that specifically target cancer hallmark processes such as hypoxia (^18^F-fluoromisonidazole) [27], proliferation (^18^F-fluorocholine, ^18^F-fluorodeoxythymidine) [28] and tumor and microenvironment acidity (pHLIP) [29, 30] may add more diagnostic, predictive and prognostic information [31] and therefore it can be expected that the full potential of PET/MRI is not realized yet.

Previous studies have suggested that larger tumor size, irregular or spiculated shape and margin, heterogeneous internal enhancement, and wash-out kinetics on DCE-MRI as described using the BI-RADS lexicon and descriptors to be most strongly indicative of malignancy [32–34], which is in good agreement with our results. According to several studies and the revised BI-RADS lexicon, the combination of morphological and functional information acquired with DCE-MRI is necessary for breast cancer diagnosis [35]. Likewise, in the present study, multivariate logistic regression analysis identified tumor enhancement kinetics to be most strongly indicative of breast cancer.

Imaging parameters of healthy breast parenchyma are currently being examined as potential non-invasive predictive and prognostic breast cancer biomarkers [14, 15, 36]. Recently, BPE has been proposed as a risk factor for breast cancer in high-risk patients who were compared with matched control groups with benign or false-positive findings [14, 15]. These initial results were confirmed by Grimm et al., who compared another 61 high risk cancer patients with matched controls [37]. Hence, it has been suggested that BPE might represent metabolic activity of breast parenchyma and, as such, an environment beneficial for cancer development [38]. Unexpectedly, our results demonstrate that decreased contralateral BPE is strongly associated with breast cancer diagnosis, while previous studies in high-risk populations have suggested otherwise [14, 15, 37]. Our findings might be the result of a stealing phenomenon of contrast agent to the breast with cancer due to higher vascularity. Furthermore, these divergent results might occur as a result of these studies focusing solely on high-risk populations whose breast tissue is known to substantially differ from women of average cancer risk [39]. In women at average risk, BPE has not been demonstrated to be a risk factor yet, as indicated by Bennani-Baiti et al. [36]. Similarly, the role of FGT with regard to cancer risk remains unclear, although it is equivalent to mammographic breast density, an established risk factor for breast cancer [38]. While King et al. demonstrated mildly increased cancer odds for patients with increased FGT in a high risk population (OR: 1.2) [14], no correlation between FGT and the presence of cancer was found by Bennani-Baiti et al. in women at average risk [36]. These findings are in good agreement with our study, where contralateral BPE is strongly predictive of breast cancer, while FGT did not add accuracy. Further research with larger cohorts of individuals at average risk is warranted to confirm our results and clarify the underlying processes.

The multiparametric PET/MRI model developed in this study identified tumor ADCmean as one of three parameters essential for breast cancer diagnosis. Our results confirm the growing body of evidence that DWI is a valuable complimentary technique to DCE-MRI, adding specificity as well as functional information [5, 20, 40–45], and hence should constitute an essential part in breast imaging. Tumor ADCmean has even been found to be potentially useful as a non-invasive biomarker for tumor grade, subtype, receptor status, and recurrence [46, 47]. A recent study found ADC values of 248 benign and malignant lesions to be independent from BPE, FGT, and menopausal status [48]. This study verifies the usefulness of lesion ADC as a stable, reliable imaging biomarker in breast imaging despite different patient characteristics. Meanwhile, apart from their positive correlation with mammographic breast density and independence of menstrual cycle and BPE [49–51], not much is known about ADC values of healthy breast parenchyma. ADC values of the healthy breast did not add accuracy to the model developed in this study.

This study has some limitations. First, BPE and FGT were assessed qualitatively. However, the BI-RADS lexicon recommends qualitative assessment of these parameters, and excellent inter- and intra-reader agreement between experienced readers has been demonstrated previously for DCE-MRI and DWI parameters including BPE and FGT [4, 16, 52, 53]. Second, our study was not performed using a hybrid PET/MRI scanner, but fused PET/CT and MRI instead. Potential benefits of using a hybrid scanner would be improved fusion of PET and MRI images, as well as acquisition of both imaging series on the same day. Hence, in our study, not all MRI and PET/CT examinations were performed on the same day (range 0–6), which might have influenced parameters such as BPE and BPU due to changes with menstrual cycle [18, 54]. Nevertheless, relevant changes in these biomarkers seem unlikely, as the mean time of 1.15 days between examinations was short. Third, in this study, the same data set was used to train and evaluate regression models. Typically, such models perform better on the data they were trained with than on additional data. Further studies with larger patient collectives, separate training and validation datasets, and bilateral quantitative assessment of all parameters are warranted to confirm our findings. In addition, the model developed in this study needs to be validated in an independent test group.

In conclusion, in a multiparametric [^18^F]FDG PET/MRI model including imaging biomarkers of the tumor and healthy breast tissue, tumor enhancement kinetics, ADCmean tumor, and BPE of the contralateral breast were most strongly associated with breast cancer diagnosis. Other imaging biomarkers did not significantly contribute to breast cancer diagnosis. Implementation of multiparametric PET/MRI imaging biomarkers of healthy breast tissue for breast cancer diagnosis should be considered.
